# Cytokine Profile of Patients with Allergic Rhinitis Caused by Pollen, Mite, and Microbial Allergen Sensitization

**DOI:** 10.1155/2017/3054217

**Published:** 2017-10-04

**Authors:** Yury A. Tyurin, Svetlana A. Lissovskaya, Rustem S. Fassahov, Ilshat G. Mustafin, Anton F. Shamsutdinov, Marina A. Shilova, Albert A. Rizvanov

**Affiliations:** ^1^Department of Biochemistry and Clinical Laboratory Diagnostics, Medical State University, Butlerov Street 49, Kazan, Republic of Tatarstan, Russia; ^2^Laboratory of Immunology and the Development of Allergens, Mycology Research Laboratory, Research Institute of Epidemiology and Microbiology, Big Red Street 67, Kazan, Republic of Tatarstan 420015, Russia; ^3^Institute of Fundamental Medicine and Biology, Kazan Federal University, Kremlevskaya Street 18, Kazan 420008, Russia; ^4^I.M. Sechenov First Moscow State Medical University, 8-2 Trubetskaya Str., Moscow 119991, Russia

## Abstract

Allergic rhinitis (AR) is especially prevalent among the population of large cities. Immunologically, the airway epithelium is a region where the population of allergen-presenting cells concentrates. These cells actively express a group of receptors of the innate immune system. A specific cytokine profile is its representation. The study was aimed at evaluating the cytokine profile in patients with seasonal and perennial allergic rhinitis. The cytokine profile of nasal secretion and blood serum of 44 patients with AR was studied. 24 of them had seasonal allergic rhinitis (SAR), and 20 patients suffered from perennial allergic rhinitis (PAR). The patients' age ranged from 4 to 60 years. It was determined in our study that the activation of the GM-CSF production retained in patients with PAR sensitized to mite allergen components (*Dermatophagoides pteronyssinus*). There was a higher production profile of TNF-*α* and TSLP in nasal secretion in the patients with perennial allergic rhinitis and additional high sensitization to SEs. Sensitization to mold fungal allergen components significantly increases in patients with seasonal allergic rhinitis. They demonstrated high level of sensitization to the *Aspergillus fumigatus* component m3. Thus, along with other clinical trials, the study performed would clarify some aspects of molecular pathogenesis of human allergic rhinitis.

## 1. Introduction

At present, allergic rhinitis is increasingly becoming an urgent problem for primary health care as the number of visits to general practitioners for this disorder rises. There is a high prevalence of allergic rhinitis (AR) especially among the population of large cities. This allergic disorder requires that therapists and allergists should perform diagnostic tests in order to confirm its diagnosis and detect underlying bronchial asthma. The incidence of allergic rhinitis and other respiratory allergies, atopic (allergic) asthma in particular, has significantly increased over the past fifty years. This is the case in most of European countries including Russia and has been attributed to environmental factors, urbanization, and changes in a diet and a lifestyle of a modern urban dweller [1].

According to recommendations provided by the ARIA expert working group in cooperation with the World Health Organization (WHO), the World Organization of Family Doctors (WONCA), and the International Primary Care Respiratory Group (IPCRG), allergic rhinitis is a chronic airway disorder and one of the most significant risk factors for the development of bronchial asthma [2].

Pathophysiologically and immunologically, the airway epithelium is a region with a concentrated population of allergen-presenting cells (APCs) called dendritic cells (DCs) which express receptors of the innate immune system. In patients with allergic rhinitis, these cells are involved in binding allergens, processing them into peptides, and presenting them via the major histocompatibility complexes MHC classes I and II for T cell receptors. The intact respiratory mucosa has been established to contain no DCs at birth; however, the exposure to biologically active aeroallergens (see Table 1) activates the respiratory epithelium.

These stimuli cause the release of chemoattractants (CCL20, CCL19, and CCL27), which initiate DC migration from bone marrow to the respiratory mucosa [3, 4]. A cytokine of GM-CSF, released by the respiratory epithelium, in cooperation with IL-4 and tumor necrosis factor-*α* (TNF-*α*) causes DC maturation. Tissue basophils are supposed to be a source of IL-4. For inhaled allergens, such as those from house dust mites, it is proposed that basophils amplify the T_H_2 immune response that is initiated by mucosal DCs in highly allergic individuals and influenced by innate immunity signaling through receptors such as Toll-like receptor 4 (TLR4) and C-type lectin signaling on epithelial cells and DCs [5]. Polarization toward a T_H_2 subtype is also under epigenetic regulation. TLRs are key components of the innate immune system that mediate immune response to PAMPs in the form of microbial, fungal, and viral products and their ligands, including endotoxins (recognized by TLR4), microbial lipoproteins (TLR2 and TLR6), viral double- and single-stranded RNA (TLR3 and TLR7/8), and bacterial CpG-containing DNA (TLR9) [6]. Other PRRs of the immune system are activated in response to endogenously generated danger signals (DAMPs) produced during allergic tissue inflammation, such as free ATP and uric acid [7]. These immune responses are also specific for allergic inflammation of the upper respiratory airways. However, the effect of relevant microbial allergen components on an immunity-mediated inflammation response character in seasonal and perennial allergic rhinitis is not studied. Its reflection is a specific cytokine profile. The profile of those cytokines such as TSLP, TNF-*α*, and GM-CSF which are synthesized in the mucosal epithelial cells, when indirectly stimulated through a system of innate immunity receptors, is of utmost interest.

The study was aimed to evaluate the cytokine profile of nasal secretion and blood serum in patients with seasonal and perennial allergic rhinitis with a potential for additional sensitization with microbial allergens.

## 2. Materials and Methods

### 2.1. Patients

The diagnosis of allergic rhinitis was established on the basis of the clinical criteria and recommendations of ARIA, 2010 [8]. The inclusion criteria were as follows: a diagnosis of allergic rhinitis for at least 2 years, specific symptoms of allergic rhinitis (an allergic rhinitis questionnaire), detected sensitization, the absence of nonallergic disorders of the nasopharynx and other organs and systems, age of patients from 4 years to 60 years, and distribution by sex in the ratio 1 : 1.2. The exclusion criteria were as follows: chronic rhinosinusitis, nasal polyps, nonallergic (infectious) rhinitis, and age below 4 and over 60 years.

#### 2.1.1. Control Group

The inclusion criteria were as follows: healthy volunteers at the age of 3–43 years without any findings of allergic disorders at examination and distribution by sex in the ratio 1 : 1.14. The exclusion criteria were as follows: chronic pathology of the nasopharynx, chronic rhinosinusitis, nasal polyposis, and age below 3 years and over 60 years.

### 2.2. Methods

#### 2.2.1. Allergy Testing

Cytology of the inferior nasal concha imprint swabs was performed. Romanowsky-Giemsa staining was used. Cells were counted with direct microscopy under a Mikmed-5 microscope (Russia) with a magnification of ×80. A percentage of epithelial cells, eosinophils, neutrophils, and lymphocytes per 100 cell elements of the stained imprint swabs was determined.

The total IgЕ concentration was evaluated with the use of an immunoassay total serum IgE kit.

Sensitization to mite, microbial, and pollen allergens was detected with an ImmunoCAP® technology based on immunofluorescence when allergens are sorbed on a 3D cellulose sponge that increases a specific antigen-binding surface. The immunoassay was carried out with the analyzer ImmunoCAP 100, v.1 (PhadiaAB, Uppsala, Sweden). Reagents for enzyme immunofluorescence assay of specific immunoglobulins (Ig) to house dust mites *Dermatophagoides pteronyssinus* (d1) and *Dermatophagoides farina* (d2) and their allergen component *Der p 1* being mite serine proteinase; to fungi *Penicillium notatum* (m1), *Cladosporium herbarum* (m2), *Aspergillus fumigatus* (m3), *Alternaria alternata* (m6), *Aspergillus terreus* (m36), *Rhizopus nigricans* (m11), and *Fusarium proliferatum* (*F. moniliforme*) (m9); to birch (*Betula verrucosa*) pollen allergens rBet v 1 and rBet v 2 (profilin, t216); to common wormwood allergens (w6); and to microbial allergens staphylococcal enterotoxin A (m80) and staphylococcal enterotoxin B (m81) were used.

### 2.3. Immunoassay

Immunoassay was performed in the clinical and diagnostic laboratory of the Kazan Research Institute of Epidemiology and Microbiology. In order to evaluate the innate and adaptive immunity, the cytokine profile of blood serum (IL-4, IL-10, and TGF-*β*) and nasal secretion (TSLP, IL-1*β*, TNF-*α*, and GM-CSF) was determined.

### 2.4. Nasal Secretion

A nasal secretion was sorbed on a cotton swab in the middle nasal concha for 30 sec, transferred into 0.25 ml of physiological saline, and centrifuged at 1500 rpm for 10 min to precipitate cellular elements, and the supernatant was collected and frozen at *T* = −20°C.

### 2.5. Cytokine Concentration Assessment

Cytokine concentrations in secretion and serum samples were determined with the use of enzyme immunoassay kits “interleukin-1 beta-EIA-BEST” (AO Vector-Best, Novosibirsk, Russia), “interleukin-4-EIA-BEST,” and “TNF-*α*-EIA-BEST” (AO Vector-Best, Novosibirsk, Russia) in accordance with the manufacturer's instructions. To determine TGF-*β*, IL-10, and GM-CSF concentrations, enzyme-linked immunosorbent assay kits were used (eBioscience, Bender MedSystems). The Human TSLP Quantikine ELISA Kit (R&D Systems, MN, USA) designed to measure human thymic stromal lymphopoietin in cell culture supernates, serum, and plasma with ELISA was used for the quantitative assessment of TSLP.

### 2.6. Statistical Analysis

The median of the parameter, standard deviation (SD), and the arithmetic mean were calculated. The nonparametric one-way ANOVA “Tukey's multiple comparison test” was applied to compare measurable characteristics between groups. The differences were considered significant at *p* < 0.05. To calculate statistical functions, the GraphPad Prism v.5 analysis program was used.

## 3. Results

### 3.1. General Characteristics of Patients with AR

Symptoms of mild and moderate rhinitis were the specific characteristic of patients with allergic rhinitis. Runny nose, nasal itching, and nasal congestion of various degrees prevailed. The characteristics of patients with AR enrolled into the study are given in Table 2.

In the study group of perennial allergic rhinitis (PAR), patients suffered from the main symptoms (sneezing, nasal congestion and impaired nasal breathing, nasal itching, and rhinorrhea) regardless of the season. The symptoms exacerbated from time to time depending upon the change of a place of habitation and exposure to initiating agents (cigarette smoke, cold air, and occupational factors). 10 patients with PAR had intermittent and mild symptoms in duration, with 10 others having persistent and moderate ones. In 5 patients, PAR was combined with other allergic pathologies (atopic asthma, atopic dermatitis).

In the study group of patients with seasonal allergic rhinitis (SAR), the main symptoms (sneezing, nasal congestion and impaired nasal breathing, nasal itching, and rhinorrhea) debuted in early summer and persisted throughout the summer to early autumn. 11 patients with CAR had intermittent and mild symptoms in duration, while 13 others had persistent and moderate ones. Seven patients with SAR had other allergic pathologies (atopic asthma, atopic dermatitis, and allergic conjunctivitis).

## 4. Sensitization Profile in SAR

12 patients with SAR were found out to have sensitization to a wormwood allergen component (w6) (Table 3).

There was sensitization to the main birch allergen component rBet v 1 in the majority of SAR patients examined (*N* = 24). One patient had an allergen-specific IgE level more than 50 kUA/l. It was a combination of SAR with persistent moderate atopic asthma. It was detected that 5 patients with SAR exhibited a high level of sensitization to allergen components of both wormwood (w6) and birch (rBet v 1).

The level of allergen-specific IgE to w6 was high ranging from 23.5 to 45.7 kUA/l, while that to rBet v 1 ranged from 5.8 to 31.8 kUA/l. It was determined that 14 patients with SAR and a high level of allergen-specific IgE to the main component, rBet v 1, had sensitization to the minor birch allergen component, rBet v 2 (profilin). An average level of allergen-specific IgE to the minor birch allergen component profilin (rBet v 2) was 2.01 kUA/l, while that of IgE to the main allergen component rBet v 1 was 23.16 kUA/l.

## 5. Sensitization Profile in PAR

The distribution of patients along the profile of sensitization to allergic components of house dust mites is presented in Table 4. Sensitization to an allergen component (d1) of the house dust mite *Dermatophagoides pteronyssinus* was detected in 15 patients with PAR. Sensitization to an allergen component (d2) of the house dust mite *Dermatophagoides farina* was detected in 14 patients with PAR. Eleven patients with PAR were detected to have a high sensitization level to two house dust mite allergen components (d1 and d2) simultaneously.

The majority of the patients (*N* = 14) with PAR examined had sensitization to the two allergen components (d1 and d2) of the house dust mites *Dermatophagoides pteronyssinus* and *Dermatophagoides farina*. At the same time, the concentration of allergen-specific IgE to the allergen component d2 was more than 89.0 kUA/l in 4 patients, 2 of them had PAR combined with atopic dermatitis and recurrent bronchitis. The concentration of allergen-specific IgE to the allergen component d1 was more than 70.0 kUA/l in 4 patients, two of whom had combined severe persistent atopic asthma and allergic conjunctivitis.

In patients with PAR, an average concentration of allergen-specific IgE was 43.37 kUA/l to the allergen component d2 of the mite *Dermatophagoides farina* and 36.9 kUA/l to d1. At the same time, the concentration of allergen-specific IgE to the minor allergen (d202) Der p 1, being cysteine protease, was 16.9 kUA/l in these patients.

## 6. Additional Sensitization to Microbial Allergens in PAR

The distribution of patients with PAR at the sensitivity profile to allergens of *S. aureus* is presented in Table 4. From the point of view of pathophysiology, we have studied sensitization to allergens (m80, m81) of *Staphylococcus aureus* in these patients. A potential of additional sensitization to microbial allergens cannot be ruled out in patients with PAR. These allergens are staphylococcal superantigens SEB and SEA.

## 7. Additional Sensitization to Fungal and *Staphylococcus aureus* Allergens in Patients with SAR

Sensitization to mold fungal allergen components was evaluated in the study group of patients with seasonal allergic rhinitis (Table 5). We have determined that the patients with SAR studied had additional sensitization to allergen components of mold fungi such as *Penicillium notatum* (m1), *Cladosporium herbarum* (m2), and *Aspergillus fumigatus* (m3).

There was sensitization to the *Staphylococcus aureus* allergen component m81 in 12 patients, while allergen-specific IgE concentrations ranged from 1.0 to 17.0 kUA/l; that is, it was moderate and high. Seven patients demonstrated sensitization to the *Staphylococcus aureus* allergen component m80, having allergen-specific IgE concentrations at the range of 2.1 to 10.2 kUA/l; that is, it was moderate and high.

## 8. TSLP Concentrations in Nasal Secretion of Patients with PAR

TSLP is a cytokine which is of great interest in allergic disorders. It has been established at present that in allergen-sensitized persons, the epithelial cells of the upper airways are capable of synthesizing TSLP where this cytokine affects DCs causing their maturation and activation. We have noticed a significant correlation (*r* = 0.46, *p* = 0.014) between the TSLP concentration in nasal secretion and that in allergen-specific antibodies (IgE) to a *Staphylococcus aureus* enterotoxin (the allergen component m80) in patients with PAR. There was a significant correlation (*r* = 0.56, *p* = 0.008) between TSLP and GM-CSF cytokine concentrations in nasal secretion of these patients.

## 9. TSLP Concentrations in Nasal Secretion of Patients with SAR

There was a significant correlation dependence between TSLP cytokine concentrations in nasal secretion and those in allergen-specific antibodies (IgE) to the allergen component m3 of the mold fungus *Aspergillus fumigatus* in the patients with SAR (*r* = 0.43, *p* = 0.023).

## 10. TNF-*α*, IL-1*β*, and GM-CSF Cytokine Concentrations in Nasal Secretion in AR

We have studied the relationship between the level of specific sensitization to allergens of mite, fungi, and bacteria in contact with the mucosa of AR patients and concentrations of cytokines, particularly TNF-*α*, IL-1*β*, and GM-CSF, in secretion. Human granulocyte macrophage colony-stimulating factor (GM-CSF) is a small glycoprotein that primarily stimulates the production and functioning of eosinophils, monocytes, and neutrophils. The GM-CSF cytokine is produced by the airway epithelial cells in an allergic inflammation. There was a significant correlation (*r* = 0.58, *p* = 0.007) between GM-CSF concentrations in nasal secretion and those in allergen-specific IgE antibodies to the allergen component d1 of the house dust mite *Dermatophagoides pteronyssinus* in patients with PAR. At the same time, a significant correlation between GM-CSF concentrations in nasal secretion and a level of allergen-specific antibodies (IgE) to the minor house dust mite allergen d202 was determined in these patients. The allergen component d202 is the cysteine protease of house dust mites.

There was a similar significant correlation dependence (*r* = 0.53, *p* = 0.014) between GM-CSF concentrations in nasal secretion and those in allergen-specific IgE to enterotoxin (m81) of *Staphylococcus aureus*.

There was also a dependence between secretion TNF-*α* concentrations and sensitization (antibody levels) to a *Staphylococcus aureus* enterotoxin (the allergen component m81) (*r* = 0.43, *p* = 0.049).

In patients with SAR, there was a significant dependence between nasal secretion concentrations of TSLP and GM-CSF (*r* = 0.54, *p* = 0.006). In patients with SAR, there was a significant dependence between GM-CSF concentrations in nasal secretion and a level of allergen-specific antibodies (IgE) to mold fungal allergen components m3 and m2 (*r* = 0.66, *p* = 0.0003 and *r* = 0.58, *p* = 0.002, resp.).

## 11. Serum Cytokine Profile in AR Patients

The exacerbation-specific serum cytokine profile of patients with AR is shown in Table 6.

It was determined that concentrations of TGF-*β*, lymphocyte Treg and T cell proinflammatory cytokine T_H_2/T_H_9, and IL-4 were almost twice as high in patients with SAR than in those with PAR. Significantly high IL-10 concentrations were observed in patients with PAR when compared to those with SAR. It should be noted that the serum level of IL-4 in patients with allergic rhinitis is 2.1–2.8 times higher than that in the control group.

In the patients with PAR, there was a negative significant dependence between serum concentrations of IL-4 and TGF-*β* (*r* = −0.49, *p* = 0.026). A negative correlation between blood serum total IgE levels and those of IL-10 (*r* = −0.63, *p* = 0.002) and TGF*β* (*r* = −0.49, *p* = 0.01) was also demonstrated in these patients.

The SAR serum cytokine profile (in a period of seasonal exacerbation) was markedly different from that of patients with PAR. There was a negative correlation dependence between the total IgE level and a patient's age (*r* = −0.51, *p* = 0.025) in SAR. There was a similar dependence between TGF-*β* levels and age (*r* = −0.45, *p* = 0.025) in this group of patients.

## 12. Discussion

Molecules of allergen components of anemophilous plants, fungal spores, house dust mites, and microbial antigens start actually contacting with the airway from the nasal mucosa, and it is in fact an active process. By interacting with IgE, sorbed via Fc*ε*RI receptors to mast cells, basophil and eosinophil allergens retain a high affinity to IgE [9]. At birth, the airways contain no DCs [3]. It is the activation of the respiratory epithelium by microbial components and irritating agents that initiates ingression of immature DCs from the bone marrow to mucosal membranes [4]. These stimuli cause the release of a number of chemoattractants, such as CCL20, CCL19, and CCL27, which direct dendritic cell migration toward the epithelium and underlying mucosa [10]. The epithelial cells of the mucosal membrane are able to modulate functions of immunocompetent cells [11]. The expression of PRRs for soluble regulatory molecules is specific for these cells, and these receptor-mediated systems can activate and produce cytokines which directly regulate a functional state of granulocytes, lymphocytes, and mononuclear phagocytes [12]. It has been established that activation of TLSP production by the epithelium during the action of aeroallergens can underlie the formation of allergic bronchial asthma in the future [13].

A significant relationship between the level of sensitization to staphylococcal superantigens (SEA) and TSLP concentrations in nasal secretion has been determined to occur in PAR. This might be due to pronounced changes in the bacteriocenosis (normal flora) of the nasal mucosa in this form of allergic rhinitis that releases gram-positive microflora superantigenic toxins. A potential for additional sensitization to microbial allergens cannot be ruled out in these patients. That was demonstrated in our study where 8 out of 20 patients had high concentrations of allergen-specific IgE antibodies to an enterotoxin. Staphylococcal superantigens might be one of the stimuli of local TSLP hyperproduction by the epithelium. There was a significant correlation between GM-CSF concentrations in nasal secretion and the intensity of sensitization to a staphylococcal enterotoxin (SEB) in the patients with perennial allergic rhinitis. Staphylococcal superantigens, SEB in particular, belong to a group of polyclonal activators of T cells that might account for increased concentrations of cytokines such as GM-CSF locally within the system of mucosal immunity. The patients with perennial allergic rhinitis and additional high sensitization to SEs demonstrated a higher TNF-*α* production profile due to macrophage and T cell activation by these toxins. With seasonal allergic rhinitis, an inflammatory response is limited in time, as a rule, by a contact season with pollen allergens and is short term. The bacteriocenosis of the nasal mucosa is not so markedly impaired in seasonal allergic rhinitis; however, sensitization of these patients to mold fungal allergen components significantly increases. There was particularly high sensitization to the *Aspergillus fumigatus* component m3. At the same time, mold fungal allergens can also stimulate a TSLP production by the epithelium. In this form of rhinitis, aeroallergen components of mold fungi can in addition lead to macrophage and T cell activation that can be observed as an increased level of factors such as TSLP and GM-CSF in the local cytokine profile of these patients. In turn, these factors can cause the maturation of dendritic cells into “inflammatory” DCs actively involved in the development and maintenance of an inflammatory response. House dust mite allergens play a significant role in sensitizing patients with perennial allergic rhinitis. A group of Acarina mites, the Pyroglifidae and Tyroglyfidae families, mainly prevails. Six main house dust mite allergen components are known; most of them were small proteins, for example, the allergen component Der p1. It was determined in our study that patients with PAR sensitized to mite allergen components (*Dermatophagoides pteronyssinus*) had the same activation of the GM-CSF production possibly by macrophages and epithelial cells.

Thus, a selective activation of TLRs on epithelial cells by bacterial, fungal, and mite allergens can enhance DC mobility within the respiratory mucosa and their invasion into the lower respiratory airways and thereby expand antigen processing. This process is mediated by the production of Т_Н_2 chemokines (CCL17 and CCL22) and cytokines (GM-CSF, TSLP) [14, 15]. Describing the serum cytokine profile especially in seasonal allergic rhinitis, one can note that immune-mediated inflammatory response of the upper respiratory airway mucosa is characterized by a more significant production of IL-4. In most cases, it occurs due to the selective diversity of Тh2 cells, which secrete a cluster of cytokines encoded on the chromosome 5q31-33, including interleukins IL-3, IL-4, IL-5, IL-9, and IL-13 and granulocyte macrophage colony-stimulating factor (GM-CSF) [16].

In seasonal allergic rhinitis, it might be due to more pronounced allergic properties of anemophilous plant pollen. Pollen of anemophilous grass, an allergen component of common wormwood (w6), and a birch pollen allergen component are capable of causing high sensitization in patients with seasonal AR. There was a high homology (more than 80%) between the birch allergen component Bet v 1 and other allergens including allergen components of timothy and wormwood. A high TGF-*β* concentration in seasonal AR is another relevant finding of our study. TGF-*β* in cooperation with IL-10 has been established to facilitate/promote the development of regulatory T cells (Тreg) [17].

We have determined that there was a negative correlation between TGF-*β* concentrations and a patient's age in seasonal rhinitis. The elder the patients, the less evident this cytokine concentration in blood serum in the development of allergic inflammation in seasonal AR. It should be noted that TGF-*β* is one of the cytokines which provide the inhibition of immune responses and the proliferation of immunocompetent cells. Thus, along with other clinical trials, the study performed would clarify some aspects of molecular pathogenesis of human allergic rhinitis.

## Figures and Tables

**Table 1:** Immunologically active aeroallergens.

Source of allergens	Identified biological activities
(1) Grass pollen	Pectate lyase, RNase, polygalacturonase, lipid transfer protein, profilin, expansin
(2) Tree pollen	Profilin, isoflavone reductase, pectin methylesterase, peptidyl-prolyl isomerase, 1,3-*β*-glucanase, calcium-binding protein, pectate lyase, superoxide dismutase
(3) Fungi	Protein disulfide isomerase, aldehyde dehydrogenase, RNase, vacuolar serine proteases, alkaline serine protease, enolase, aspartate proteases, dipeptidyl peptidase, subtilisin-like protease
(4) Epidermal allergens	Uteroglobin-like protein, cystatin, lipocalin, albumin
(5) House dust mites	Cysteine protease, *β*-glucan moiety, trypsin, amylase, chymotrypsin, chitinase, collagenase, glutathione transferase

**Table 2:** Characteristics of patients with allergic rhinitis.

Parameter	Form of AR	
	PAR	SAR
Number of patients, *N*	20	24

Age, years^∗^	27.8 ± 4.0 17.9 (4–60)	18.2 ± 2.86 14.01 (3–43)

Gender (M/F)	8/12	12/12

Serum total IgE level, IU/ml^∗^	100.65 ± 20.48 91.63 (14–412)	92.3 ± 6.91 33.86 (45–212)

Blood eosinophilia, %	4.91 ± 0.99 3.44 (2–12)	6.33 ± 1.08 3.7 (1–12)

Eosinophil portion in nasal mucosa imprint swab, %^∗^	27.65 ± 4.54 20.34 (3–80)	19.5 ± 3.32^∗∗^ 16.3 (2–57)

^∗^M ± m, SD: standard dev., Xmin–Xmax; ^∗∗^*p* < 0.05.

**Table 3:** Distribution of patients with seasonal allergic rhinitis (SAR) according to the level of sensitization to pollen allergen components of *Artemisia vulgaris* and birch (*N* = 24).

Level of IgE (sensitization), kUA/l		Allergen components		
	w6	rBet v 1	w6 + rBet v 1	rBet v 2
0.7–3.5 (very low)	1	2	—	14
3.6–17.5 (sensitization revealed)	4	9	—	—
17.6–50.0 (high level of sensitization)	7	11	5	—
50–100.0 (very high)	0	1	—	—
Total	12 (50.0%)	23 (96.0%)	5 (21.0%)	14 (58.0%)

**Table 4:** The distribution of patients with PAR in terms of the level of sensitization to allergen components of house dust mites and bacterial microflora (*N* = 20).

Level of IgE (sensitization), kUA/l		Allergen components		
	House dust mite			Bacteria
	d1	d2	d1 + d2	m81
0.3–0.69 (very low)	—	—	—	1
0.7–3.5 (low)	2	1	—	2
3.6–17.5 (sensitization revealed)	3	4	10	
17.6–50.0 (high level of sensitization)	6	5	—	5
50–100.0 (very high)	4	4	4	—
Total	15 (75.0%)	14 (70.0%)	14 (70.0%)	8 (40.0%)

**Table 5:** Distribution of patients with seasonal allergic rhinitis according to the level of sensitization to allergen components of mold fungi (*N* = 24).

Level of IgE (sensitization), kUA/l	Allergen components		
	m1	m2	m3
0.3–0.69 (very low)	—	—	—
0.7–3.5 (low)	3	—	1
3.6–17.5 (sensitization revealed)	—	4	—
17.6–50.0 (high level of sensitization)	—	—	3
50–100.0 (very high)	—	—	—
Total	3 (12.5%)	4 (16.7%)	4 (16.7%)

**Table 6:** Serum cytokines in allergic rhinitis.

Form of AR	Cytokine concentration, pg/ml^1^		
	IL-4^2^	IL-10	TGF-*β*^3^
SAR (*n* = 24)	0.54 ± 0.03 0.17 (0.21–0.9)	0.36 ± 0.04 0.22 (0.11–0.87)	35,900.0 ± 471.0^∗∗^ 23,090 (600.0–89,000.0)

PAR (*n* = 20)	0.4 ± 0.05 0.24 (0.11–0.87)	0.42 ± 0.04^∗^ 0.17 (0.11–0.78)	17,000.0 ± 306.0 13,700 (1700–45,008)

Control (*n* = 15)	0.19 ± 0.05 0.17 (0.1–0.34)	0.41 ± 0.04 0.22 (0–0.5)	40,300 ± 17,700 17,000 (11,000–51,000)

^1^Data are presented as M ± m, SD, and Xmin–Xmax. ^2^Differences between patients with allergic rhinitis and control group are significant (SAR versus control, PAR versus control), ^∗^*p* < 0.05. ^3^Differences between groups of patients (SAR versus PAR, PAR versus control), ^∗∗^*p* < 0.01.
